# Understanding chaos in COVID-19 and its relationship to stringency index: Applications to large-scale and granular level prediction models

**DOI:** 10.1371/journal.pone.0268023

**Published:** 2022-06-08

**Authors:** Imee V. Necesito, John Mark S. Velasco, Jaewon Jung, Young Hye Bae, Jun Hyeong Lee, Soo Jun Kim, Hung Soo Kim

**Affiliations:** 1 Department of Civil Engineering, Inha University, Incheon, South Korea; 2 Institute of Molecular Biology and Biotechnology, National Institutes of Health, University of the Philippines, Manila, Philippines; 3 Department of Clinical Epidemiology, College of Medicine, University of the Philippines, Manila, Philippines; 4 Department of Hydro Science and Engineering Research, Korea Institute of Civil Engineering and Building Technology, Gyeonggi-do, South Korea; Universiti Malaysia Sabah, MALAYSIA

## Abstract

Understanding the underlying and unpredictable dynamics of the COVID-19 pandemic is important. We supplemented the findings of Jones and Strigul (2020) and described the chaotic behavior of COVID-19 using state space plots which depicted the changes in asymptotic behavior and trajectory brought about by the increase or decrease in the number of cases which resulted from the easing or tightening of restrictions and other non-pharmaceutical interventions instituted by governments as represented by the country’s stringency index (SI). We used COVID-19 country-wide case count data and analyzed it using convergent cross-mapping (CCM) and found that the SI influence on COVID-19 case counts is high in almost all the countries considered. When we utilized finer granular geographical data (‘barangay’ or village level COVID-19 case counts in the Philippines), the effects of SI were reduced as the population density increased. The authors believe that the knowledge of the chaotic behavior of COVID-19 and the effects of population density as applied to finer granular geographical data has the potential to generate more accurate COVID-19 non-linear prediction models. This could be used at the local government level to guide strategic and highly targeted COVID-19 policies which are favorable to public health systems but with limited impact to the economy.

## Introduction

As of 28 September 2021, 290 million COVID-19 cases worldwide with 4.55 million deaths have been recorded [1]. Despite travel restrictions and other non-pharmaceutical interventions (NPIs), many countries still show sustained transmission of the virus [2] with several global waves brought on by predominant SARS-CoV-2 variants of concern (VOC). Other than the impact on public health, the pandemic and the lockdowns implemented to contain the pandemic have also significantly impacted the global economy [3] with significant contraction of the gross domestic product (GDP) observed in almost all countries in 2020 [4]. The Philippines, despite implementing one of the harshest and longest lockdowns in the world, still has not managed to contain COVID-19, with 2.5 million cases and more than 37,000 deaths recorded [5] as of 28 September 2021. In Southeast Asia, the Philippines has the second-highest number of COVID-19 cases and deaths after Indonesia [6]. The Philippines’ GDP also contracted by 9.5% in 2020 which is its worst economic performance since after World War II [7]. To minimize the impact on the economy, the Philippine government has shifted to a system of granular lockdowns at the barangay or village level [8] vis-à-vis previous wide-scale lockdowns which were imposed over entire regions or provinces. This shift has highlighted the need to develop mathematical models which can still make accurate predictions despite the decrease in the number of cases being used by the model. Creating and validating a working mathematical model which can sufficiently simulate the number of cases expected not only at the country level but also at the village or barangay level will be essential for use by the local government units in guiding the implementation of granular lockdowns. In this paper, we will try to apply chaos theory and understand the chaotic behavior of COVID-19 cases as well as its causal relationship to the stringency index (SI) and applying state space reconstruction (SSR) and Convergent Cross-mapping (CCM) to COVID-19 to selected countries at the country-wide level and using finer, granular geographical units (i.e., barangay / village).

## Materials and methods

### COVID-19 and chaos

Chaos is said to be deterministic if the course of movement is sensitive to the initial conditions [9]. Apparently, there are many systems that illustrate random and unpredictable behavior and usually, behind that randomness is an underlying order that is not easily discernible. Understanding the different forces at work in a particular system is valuable in order to know the trajectory of a system. Just like in deterministic chaos, a tiny change in initial conditions of the disease spread can have a significant effect on the course of the potential outbreak. Jones and Strigul (2020) [10] proved that the COVID-19 epidemic has demonstrated a chaotic behavior since it met all the possible criteria established by Poincaré: (1) presence of large number of solutions; (2) pattern sensitivity; (3) unpredictability; and (4) being deterministic. This paper will try to supplement their findings by showing the reconstructed phase space plots and how the plots are affected by the government-employed stringencies by applying convergent-cross mapping. The methods will be applied at the country-wide level and at finer granular geographical level using data from five (5) barangays which belong to the two (2) major cities in the National Capital Region (NCR), Philippines, namely Pasig and Quezon City. For the country-wide level, COVID-19 daily cases from 21 January 2020 to 31 December 2020 from a combination of countries recognized by CDC (as of 2021) as Very High Risk (United Kingdom, Malaysia and Brunei), High Risk (Indonesia, Japan, Philippines and Singapore), Moderate Risk (Cambodia and South Korea) and Low Risk (China) were used (CDC, 2021a). (NOTE: The CDC COVID-19 RISK country inclusions may differ from 2020 depending on the case count every 28 days) [11]. In order to avoid the interference from the worldwide roll out of SARS-CoV-2 vaccines, only 2020 COVID-19 data was used.

### Reconstruction of state space

Takens (1981) [12] has illustrated that a scalar time series without noise can be reconstructed in a multi-dimensional state space. The reconstructed state vector is represented by Eq 1 [13]:

$$x_{k}^{m}={\left[x_{k,}\;x_{k+\rho ,}\ldots x_{k+(m-1)\rho }\right]}^{T}$$ (1)

where k = 1,…, N, T> 0, m is the embedding dimension, ρ is a function of time delay, τ. In this study we used the tool box of recurrence plot [14] using MATLAB R2019a. Since the theorem of Taken et al (1981) [12] considers eliminating noise before performing a state space reconstruction (SSR), this study has performed discrete wavelet transform (DWT) as a noise-elimination method [15]. Necesito et al. (2021) [16] used DWT to smoothen and detect surge boundaries of dengue incidence in the Philippines. According to Radhakrishnan (2018) [17], in analyzing multi-resolution time series or signals, the time series or signal can be divided into two parts: an “approximation” part and a “detailed” part. The scaling function which gives the approximation coefficients (*a*_*j*,*k*_) is denoted by *φ*_*j*,*k*_(*t*) and the wavelet function which gives the detailed coefficients (*d*_*j*,*k*_) is denoted by *ψ*_*j*,*k*_(*t*), where j and k are integers. DWT can be implemented using Eq 2:

$$x(t)=\sum_{k=-\infty }^{\infty }a_{j_{0,}k}\varphi_{j_{0,}k}(t)+\sum_{k=-\infty }^{\infty }d_{j_{,}k}\varphi_{j_{,}k}(t)$$ (2)

**Fig 1:**
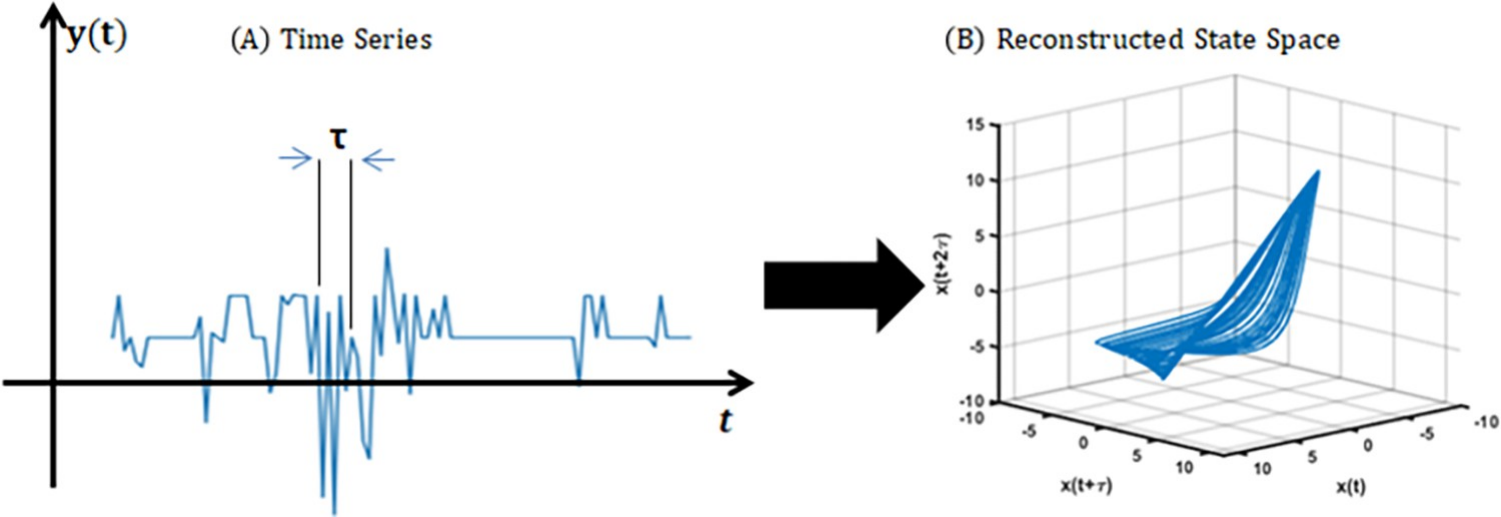
State Space Reconstruction (SSR) concept (τ = time delay).

We utilized the approximation part of the *pywt package* in Python 3.7. In this study, we used DWT to smoothen and process the COVID-19 case signals and applied SSR. Fig 1 shows the generation of the plot of state space of COVID-19 cases.

### Stringency Index (SI)

The data is collected from a publicly available database (www.ourworldindata.org). Based on the database, the formula represented by Eq (2) was used for the calculation of indices:

$$Stringency\;Index=\frac{1}{k}\sum_{j=1}^{k}I_{j}$$ (2)

According to OxCGRT, *k* represents the number of component indicators in each index, *j* is the indicator and *I* is the sub-index score. SI is a measure of response metrics in terms of school closures, workplace closures, cancellation of public events, restrictions on public gatherings, closures of public transport, stay-at-home requirements, public information campaigns, restrictions on internal movements and international travel controls [18]. This study used SSR and SI to explore the behavior of COVID-19 cases in relation to the change in restrictions in different countries, namely, Brunei, Cambodia, China, the United States (US), United Kingdom (UK), Malaysia, Japan, South Korea, Indonesia, Singapore, and the Philippines.

### Convergent Cross-Mapping (CCM)

**Fig 2:**
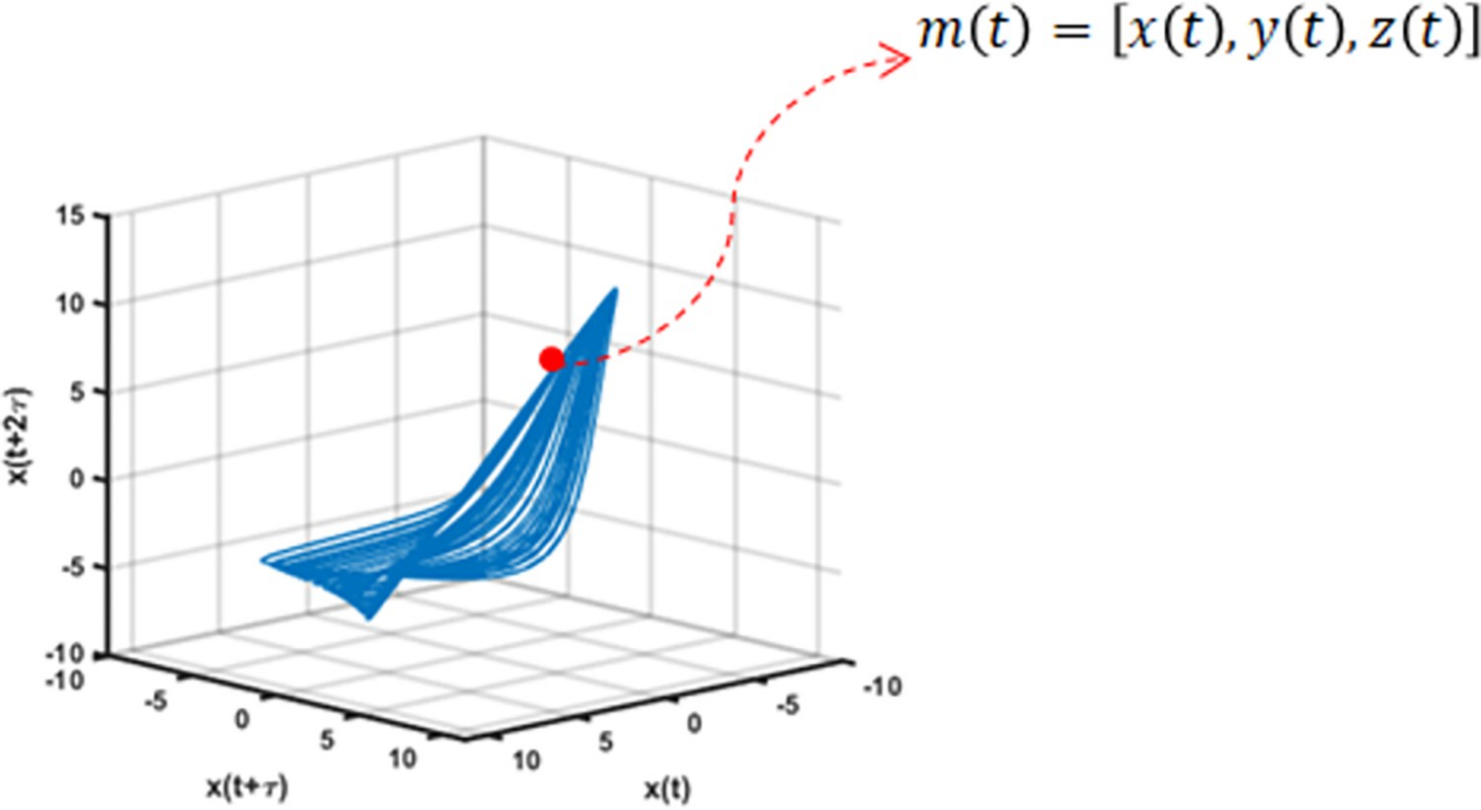
State space plot and sample coordinates.

**Fig 3:**
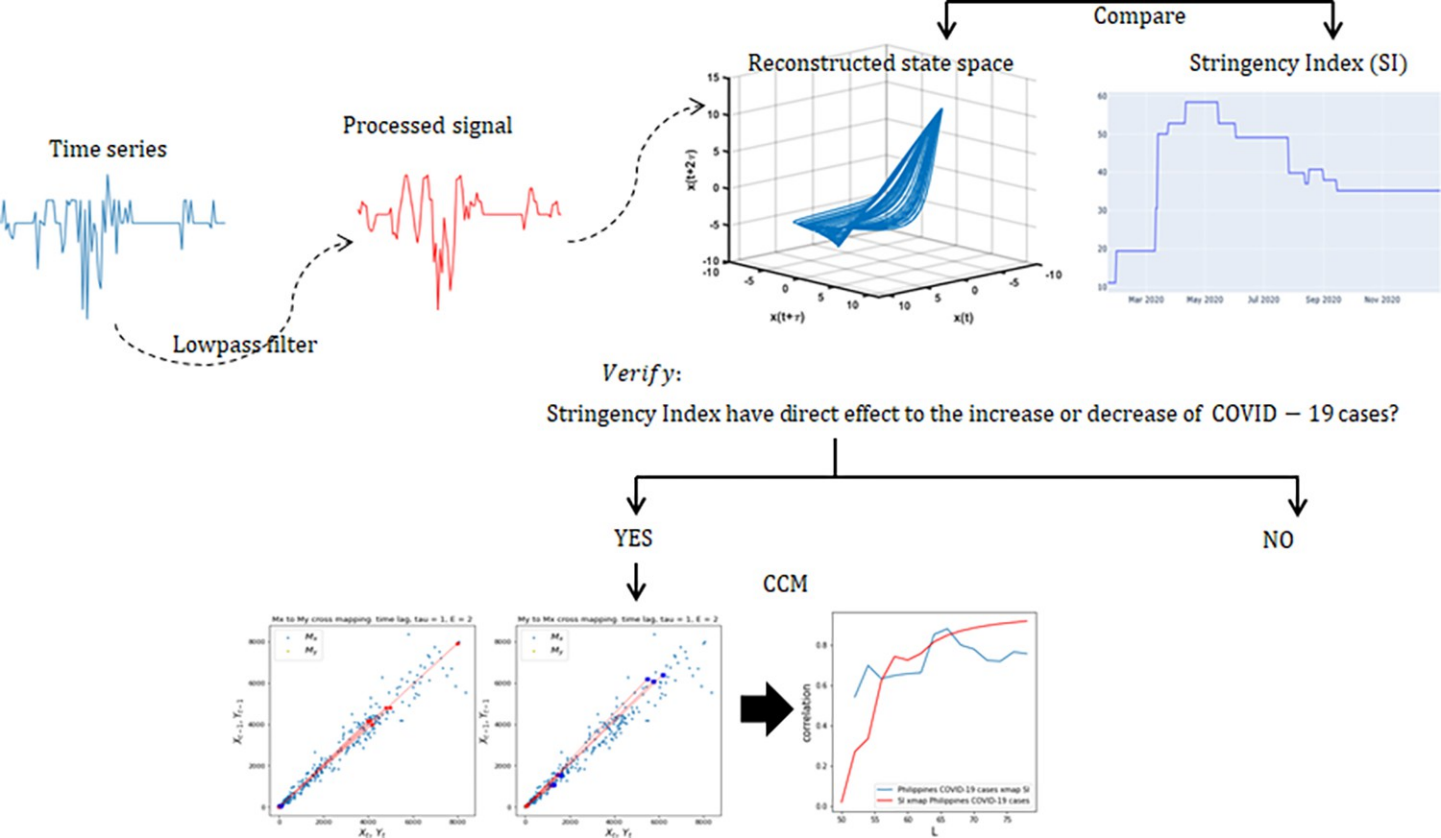
Schematic diagram in exploring COVID-19 cases.

Finding causal relationships and interactions among variables in the complex systems is a valuable aspect for evidenced-based studies such as those concerning disease prevention and public health [19]. The methodology for CCM was discussed by Sugihara et al. (2012) and claims that the method is useful when granger causality [20] is not appropriate to use. In this study, we used CCM to infer the causality between COVID-19 cases and SI using the Philippines granular geographical time series data of 5 barangays from two major cities in the National Capital Region (Pasig and Quezon City). Fig 2 shows sample state space coordinates. Eqs (3) and (4) are the new coordinates after the application of CCM. Fig 3 shows the schematic diagram of the methodology used in this study.

The causal relationship in complex systems as well as its interactions among variables is very useful for disease prevention and public health [19]. Causation can infer correlation but correlation does not essentially suggest causation. This paper utilized Convergent Cross-Mapping (CCM) to infer the causality between COVID-19 cases and SI using the Philippines granular geographical time series data of 5 barangays from two major cities in the National Capital Region (Pasig and Quezon City). As pointed out by Sugihara et al. (2012) [21], CCM can be used for non-separable systems or frameworks where the predictability of a variable, say Y, isn’t autonomously unique to another variable, say X. Hence, CCM may be a more appropriate approach for dynamic frameworks and can moreover recognize interactions among frameworks from shared factors.

Sugihara et al. (2012) [21] emphasized that CCM can test causation for a dynamic framework that’s not totally arbitrary and can recognize the correspondence between states. In his paper, he used Lorenz system with two shadow manifolds or low dimensional representation of the entire system (M_x_ and M_y_, see Eqs 3 and 4) built using lagged-coordinate embedding (τ = lag). *L* is the time series length and τ is the lag time in which can be assigned arbitrarily.

$$M_{x}:\;x(t)=[x(t),\;x(t-\tau ),\;x(t-2\tau )]$$ (3)

$$M_{y}:\;y(t)=[y(t),\;y(t-\tau ),\;y(t-2\tau )]$$ (4)

where *m(t)* is a point in the manifold, *M*, and *X* is an observation function in a temporal flow. For each function, *X*, there is a corresponding time series in which we can denote as *{X} = {X(1)……*..*X(L)}* that can track the trajectory of the points within the manifold, *M*.

On the other hand, using *x(t)* as points in the manifold, say *M*_*x*_ (which was built through the use of the time lagged values of *X*), and which *x(t)* consists of vectors such as *X(t)*, *X(t- τ)*, *X(T-2 τ)* up to *X(t-(E-1) τ)* with *E* as the dimensional state space and *τ* as the lag time, we can say that *x(t)* on *M*_*x*_ can map *m(t)* on *M*. In this way, if there are two dynamically coupled variables, say *X* and *Y*, their manifolds *M*_*x*_ and *M*_*y*_ can eventually map each other. This is due to the fact that they are a diffeomorphic reconstruction of their common manifold, *M*. The *causal-ccm package* in python [22] was used in this study.

## Results

**Fig 4:**
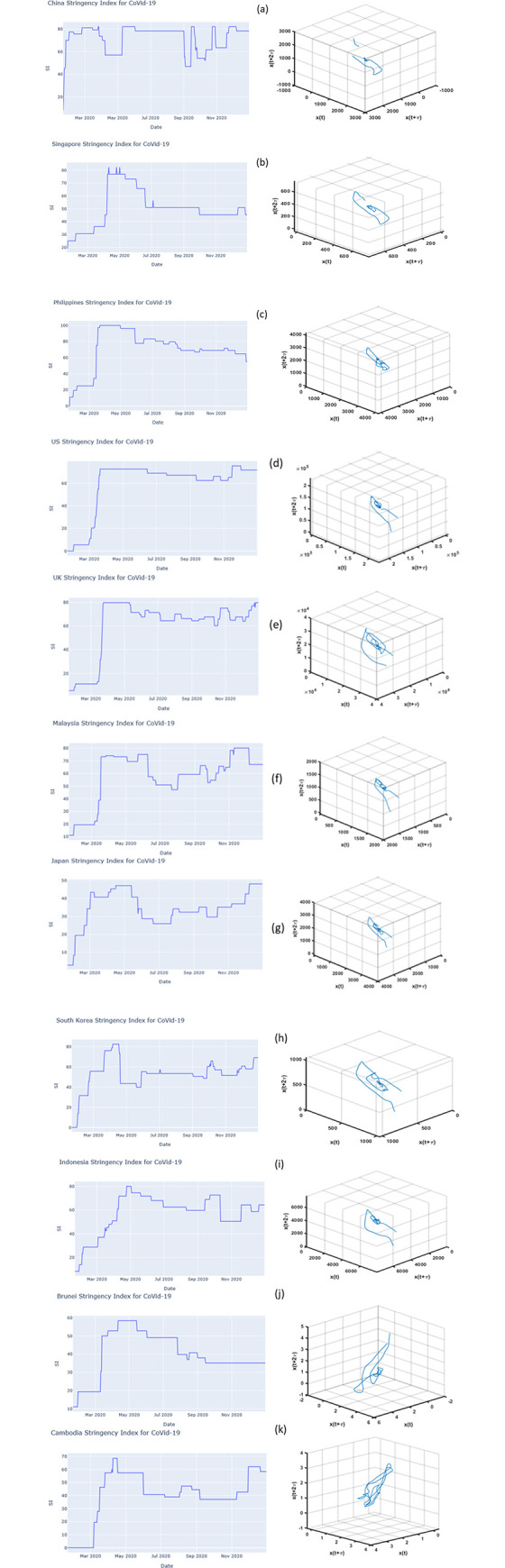
SSI and SSR of (a) China; (b) Singapore; (c) the Philippines; (d) the US; (e) UK; (f) Malaysia; (g) Japan; (h) South Korea; and (i) Indonesia; (j) Brunei; (k) Cambodia.

The state space plot for each country is shown in Fig 4. Brunei showed a combination of small and large oscillations where small oscillations meant that the number of cases for each day were not jumping from a lower to a higher number. On the other hand, Cambodia showed a consistent oscillation size which meant that during the considered period (22 January 2020 to 31 December 2020), the number of COVID-19 cases was practically stable with no noticeable surges. China’s state-space plot showed a very small oscillation frequency before turning into a larger oscillating frequency with the small oscillations attributed to the surge of cases in January 2020 and the larger oscillation due to the abrupt decrease in the number of cases. This can be interpreted as the occurrence of high initial transmissions due to large outbreaks and then a sudden decrease in the transmission rate brought about by mass testing and very strict movement measures implemented by the Chinese government [23]. In fact, according to the World Health Organization (WHO) the outbreak was declared as early as January 1, 2020 [24] and abrupt changes in the number of cases (1,777 cases on February 18, followed by 400+ cases for 3 consecutive days, and a jump of 1,451 cases on February 22 and 21 cases on February 23). On 26 February 2020, as low as 412 new cases were reported in the Situation Report of WHO [25]. These abrupt changes may also be due to underreported cases cited by some news agencies [26, 27]. As shown in Fig 4, despite the differences in their initial conditions, the US, UK, Malaysia, and Japan as well as South Korea and Indonesia appeared to have the same asymptotic behavior or observed trajectory and attractor (a point where the system tends to evolve or attract) which creates almost the same oscillation patterns while Singapore and the Philippines appeared to belong to another group. The evolution or attraction will help in determining the limiting behavior of the time series and could aid in the prediction of nonlinear systems. Looking at the state space plot of the Philippines, a trajectory like Singapore was observed. On the other hand, China showed discontinuous oscillation patterns compared to the previously mentioned countries while Brunei and Cambodia were observed to have a different asymptotic behavior or attractor compared to the others. Cambodia showed a consistent or almost uniform oscillation size for COVID-19 cases in 2020, while the US, UK, Malaysia, Japan, South Korea, and Indonesia had almost the same type of oscillation patterns (no signs of spherical or ellipsoidal pattern, but small oscillations which eventually changes to unstable larger oscillations) with more similarity with the US, UK, Japan, and Malaysia.

By visual comparison, US and UK SI and state space plots resembled each other (stringency indices from low to a steep increase then an almost stable index throughout the year 2020—except for UK where SIs were varying, with state space plots of short distances of oscillations at the center), the same goes with the SI and state space plots of Japan, Malaysia, South Korea and Indonesia. The SIs of Japan and Malaysia varied graphically with a small resemblance to Indonesia. In Indonesia, the SIs gradually decreased from May 2020 to September 2020. South Korea had larger oscillations and increasing SIs which were correlated with a sudden easing of restrictions on May 2020. Singapore’s SI and that of the Philippines showed almost the same features but since there was no significant easing of restrictions, there was not much gradual increase or decrease of SIs. China, on the other hand, was the odd one out, and showed discontinuous state space plot and gradual increase and decrease of SIs. If the SSR showed a continuous curve, it signified a high degree of determinism [28]. However, this is not the case of China’s SSR where an irregular or discontinuous output curve is apparent. This irregular or discontinuous output could be due to the introduction of random factors that affect COVID-19 transmission. Some factors affecting COVID-19 deaths and transmissions are pointed out by Roy and Ghosh (2020) [29] which include airport traffic and population density and testing, individual physiology, and pre-existing conditions to name a few.

**Fig 5:**
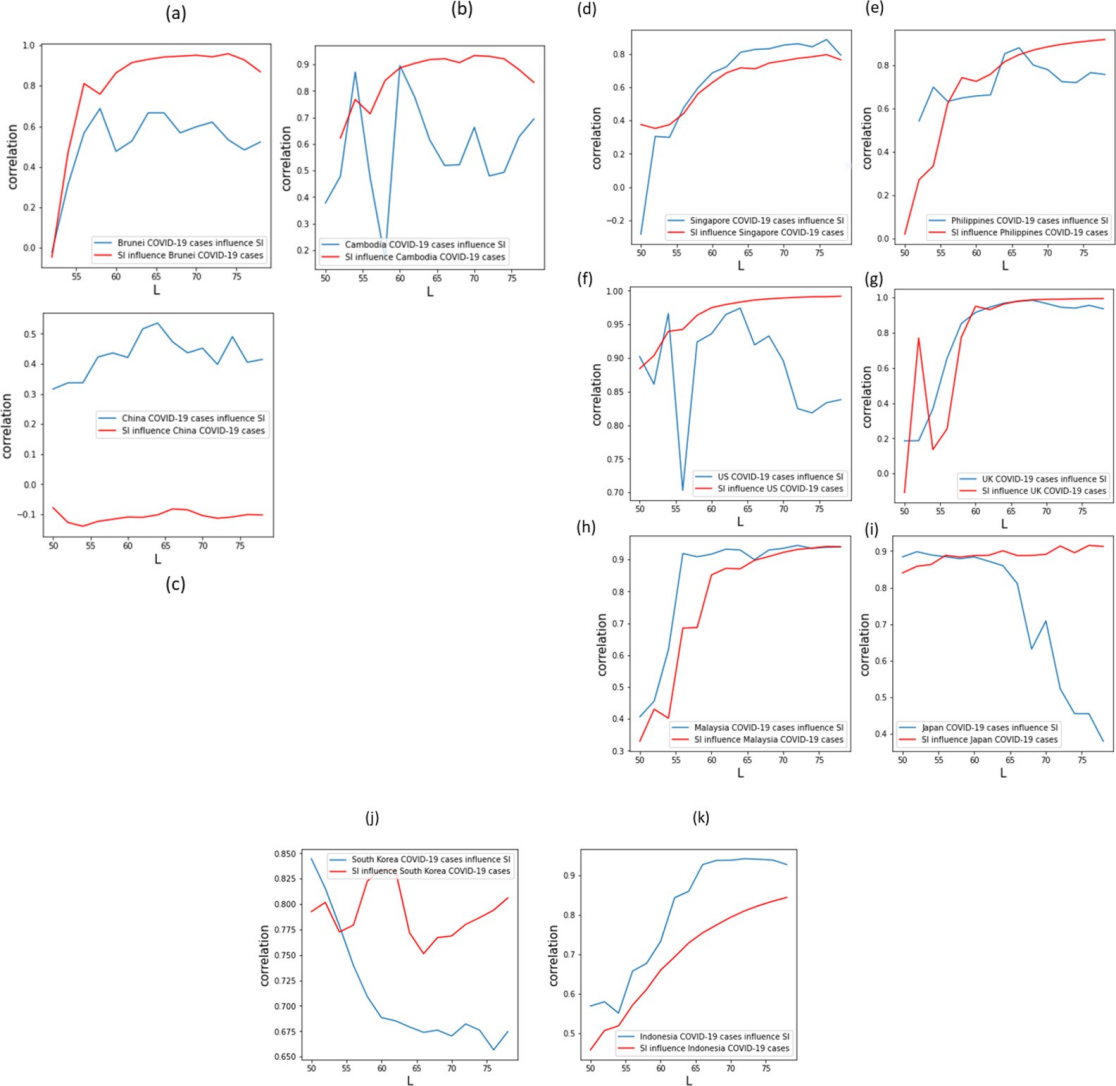
CCM results for COVID-19 and SI showing correlation on the y-axis and L as the time series length.

To be able to determine the gravity of the effect of the SIs to COVID-19 cases, CCM was employed. Fig 5 shows the CCM of COVID-19 cases versus SI of Brunei, Cambodia, China, Singapore, Philippines, the US, UK, Malaysia, Japan, South Korea, and Indonesia. For SI cross-mapping the COVID-19 cases (red) in Brunei was 0.96, Cambodia at 0.91, Singapore at 0.78, the Philippines at 0.87, 0.96 for US and UK, 0.91 for Malaysia and Japan and 0.82 for Indonesia. However, South Korea reached 0.83 followed by a downward trend before reaching 0.80 again. China, on the other hand, remained the odd one out for showing very low correlation values which imply that SI had little to no effect in the increase or decrease of COVID-19 cases. As pointed out earlier, issues of underreporting of cases which could be the cause of abrupt changes in case count can also be a contributory factor for China’s low correlation.

## Discussion

The big or small oscillations represent fluctuations of the number of cases which can be caused by external forces. As identified by Roy and Ghosh (2020) [29] in their paper, it can be social, economic, environmental, or even demographic factors which affect the number of COVID-19 infections. In the case of China, there is a big gap between the series of high number of cases and the series of low number of cases that’s why the oscillations are farther apart.

Malaysia experienced a sudden increase of active cases, thus preventive measures were imposed in mid-March of 2020. When Japan’s COVID-19 time series was converted to state space, the movement of the oscillation was not far from the center. This means that the number of cases did not jump significantly far from the previous counts. The four countries (US, UK, Japan, and Malaysia) with the same pattern had similar patterns of jumps of cases especially in the mid to the latter part of the year (August to November 2020).

Indonesia’s state space plot showed a big oscillation with smaller ones maintained in the middle. China’s state-space plot showed a very small oscillation frequency before transforming into a larger oscillating frequency which reflected China’s response which included, massive testing, and strict and active tracing of contacts in combination with risk-based easing of restrictions [30]. The jump of cases had lowered starting March 2020. As of this writing, China is considered as a low-risk country for COVID-19 [2].

Knowing attractors and the patterns of oscillations would help in determining how the system will evolve through time. The trajectories and oscillations shown by US, UK, Malaysia, and Japan can give us an idea on the external factors common to those countries which contributed to the increase or decrease of oscillations patterns. In the paper of Cross et al. (2020) [31] where the stringency index of different countries such as the US, UK and Japan were investigated, they concluded that COVID-19 infection rates were affected by the timing of the government’s response. The three countries showed 48 to 100 days of response escalation (as defined in the paper, it is the time taken to reach the maximum SI after the initial response).

South Korea’s varying oscillation pattern can be attributed to surges in cases and successful containment and subsequent flattening of COVID-19 case curves. Indonesia initially had consistently small number of cases but eventually reached a consistent surge of cases. The Brunei pattern, which appeared to be more random had almost zero case counts throughout the year 2020 and increases in the number of COVID-19 cases detected but not exceeding 30 cases at any given time.

Singapore and Philippines which both illustrated an almost similar oscillation pattern, experienced surges which can be shown in the large and small patterns of oscillation, but cases were lower for the rest of the year 2020. Singapore is considered as one of the countries with a low risk of contracting COVID-19 [2]. The country’s response involved extensive testing with 57,245, tests per million population in addition to its contact tracing which made use of their well-established, technology-based, centrally controlled surveillance system, lockdowns, and other social distancing measures [32]. The success of Singapore’s response to the virus spread was shown by the consistently small oscillation patterns.

Looking at the state space plot of the Philippines, there are points in the trajectory which were far from the previous trajectories. These are cases which oscillate far from previous case counts such as what was observed in August 2020. Both Singapore and Philippines showed similar oscillation patterns around July and beginning of August 2020, with a sudden surge recorded on September 2020.

Chaos theory makes use of oscillation patterns instead of case count time series for predictability, however, this paper will not focus on the chaos nonlinear prediction of the COVID-19 transmission. As pointed out, by Jones and Strigul (2020) [10], there is a varying model behavior among different countries and so are the potential model solutions that could aid its predictability. This is supplemented by the small and large oscillation sizes of the plots of state space in the countries presented in this study. Because of the different possible solutions and behavior of COVID-19, another criterion, which is unpredictability, was also confirmed. Different oscillation shapes were found especially in Brunei and Cambodia which indicated the need for a different solution or model approach compared to other countries.

To find out whether the COVID-19 pandemic met the other criteria mentioned by Jones and Strigul (2020) [10], SI of the Oxford Coronavirus Government Response Tracker (OxCGRT) (www.ourworldindata.org) was obtained (Fig 4). The blue lines (COVID-19 cases cross-mapping SI) showed increasing-decreasing patterns of correlation which were observed in almost all the countries except Singapore, UK, Malaysia and Indonesia. On the other hand, Japan and South Korea showed decreasing patterns. The high correlation of both convergent cross-maps of almost all the countries showed a strong coupling or bond between the two variables. This confirms that SI had effects on the number of COVID-19 cases and that it can be used to predict COVID-19 cases. Knowing this information, it is possible that using SI as a potential variable for prediction, all tested and untested or detected and undetected, symptomatic, and asymptomatic patients can also be included in the non-linear prediction model. According to the Centers for Disease Control and Prevention (CDC), people with symptoms and are potentially exposed to the infected person/s should be tested [33]. However, low-income countries (e.g. Philippines) whose resources are limited may have difficulty in testing a massive number of people in addition to tracing the contact person/s of the infected patient/s [34]. This accounts for the aforementioned untested or undetected symptomatic or asymptomatic patient/s.

The results of the CCM also confirm the two criteria as stated by [10] for chaos, which is sensitivity and deterministic property. Sensitivity, since the varying oscillations are affected by the rise or drop of SI per country and deterministic property of a chaotic system since it implies that there are factors that could contribute to the spread of the disease. Due to high CCM correlations, SI can be considered as one of the variables which can be included in future prediction models for COVID-19.

### Application of CCM to a granular geographical level

CCM was also applied to a more granular geographical time series data of the Philippines. ‘barangay’ or village is the smallest political and implementing unit for government policies and programs in the Philippines. Using 2020 COVID-19 data of five (5) barangays in the Philippines and the country’s SI from 1 September 2020 to 31 December 2020 (COVID-19 case counts categorized per barangay are available starting in September 2020 only), CCM was applied to understand how SI affects the number of COVID-19 cases (and vice versa) at a finer geographical level. Two major cities in the NCR were considered. From them, five (5) ‘barangays’ with varying population densities were chosen for this study.

**Fig 6:**
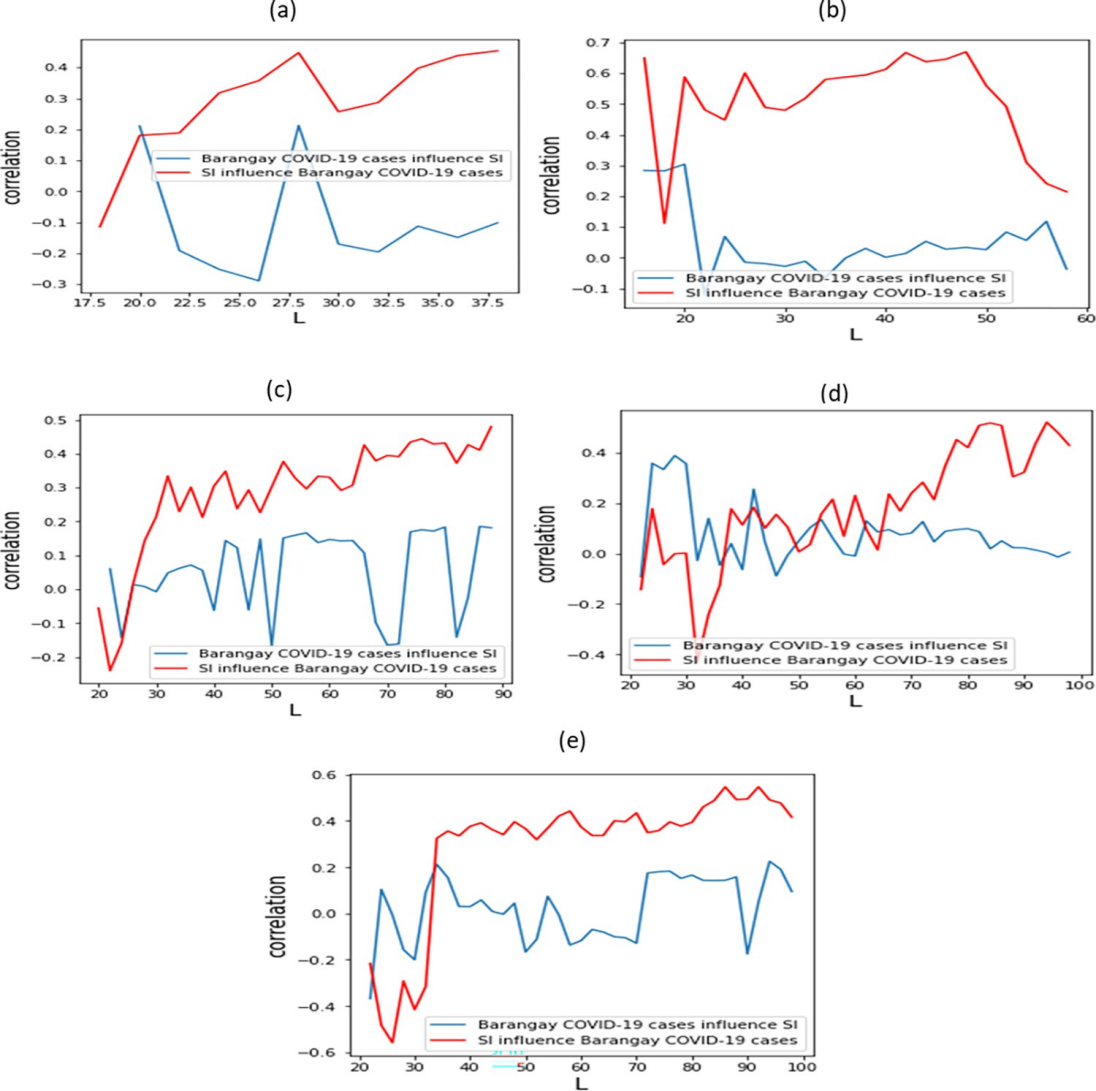
CCM applied in a granular administrative level in the following barangays (a) Oranbo, Pasig City; (b) Dela Paz, Pasig City; (c) Caniogan, Pasig City; (d) Payatas, Quezon City; and (e) Commonwealth, Quezon City.

In Fig 6, the results of CCM of SI values to the COVID-19 cases of different barangays in order of increasing population density showed consistent results: the SI showed increasing influence or impact to the number of COVID-19 cases in the community. On the other hand, COVID-19 cases influencing SI showed almost a stable trend in all the studied barangays (with very low correlation value not exceeding 0.4). This could imply two things: (1) inefficient implementation of intervention policies; and (2) intervention policies were not strictly followed by the bounded population. We observed that correlation of the SI cross-mapping the COVID-19 cases seemed to be influenced by population density such as in the case for (a) Oranbo (6,268/km^2^), (b) Dela Paz (15,075/km^2^) and (c) Caniogan (26,903/km^2^) barangays which had a correlation of 0.43, 0.50 and 0.7 respectively. The most densely populated barangay, (d) Payatas in Quezon City, had a population density of 44,390/km^2^ and which had a 0.56-high of correlation while (e) Commonwealth barangay which had a population density of 59,721/km^2^, had a correlation of 0.49 which depicts that as the population density increases, the impact of SI decreases.

The results can be used to describe COVID-19 micromanagement at the barangay level in the two cities of Pasig and Quezon City. The red curves in Fig 6, which represent how SI affects or cross-maps the COVID-19 cases of the three barangays in Pasig City (Oranbo, Dela Paz and Caniogan), show shallow drops or changes as compared to the high density population of Quezon City (Payatas and Commonwealth) which show larger changes or drops. This could indicate that in barangays with high population density, effects of SI are reduced. Granular lockdowns with imposition of varying SI interventions depending on the population density—can also be a potential alternative. Fig 6(D) and 6(E) showed a 0.2 change while low-density barangays of Pasig (Fig 6A to 6C) showed changes of 0.1 or less.

## Conclusion

This study has supplemented the findings of Jones and Strigul (2020) [10] about the chaotic behavior of COVID-19 by showing the SSR of COVID-19 case counts with emphasis on the oscillations changes or trajectories of case counts which the authors associate with the ease or tightening of restrictions as represented by the country’s SI. We observed a high SI correlation in majority of the COVID-19 country-wide analysis (Brunei, Cambodia, Singapore, Philippines, the US, UK, Malaysia, Japan, South Korea and Indonesia) and the analysis done at the granular (barangay/village) level. By analyzing the latter (granular lockdowns in the Philippines), we found that SI effects were reduced when the population density increased. Since many countries have been economically suffering due to the disruption of supply chains caused by the imposition of hard lockdowns [35], a revised policy considering the overlapping situations of economic and social health should be imposed especially in countries where GDP is highly affected by COVID-19.

This study had several limitations. We were not able to target the SI threshold value appropriate for each country included in the analysis. Breaking down SI to more precise variable indices specific to a granular geographical area rather than using SI in general may result to a more accurate prediction model. Despite finding a causal relationship between COVID-19 cases and SI which can be a good starting point for COVID-19 prediction modeling and policy decision making, we should emphasize that SI is just a numerical index which may not directly translate to actual implementation or compliance of the population to the suggested interventions. For example, even if the reported SI is high but if the implementation does not equate to the level of the SI (especially in areas where the population is high and therefore difficult to micromanage), the potential impact of the interventions on the local outbreak will be significantly mitigated. Another possibility is that the population bounded by SI may not be strictly following the rules imposed by the local governments. Such factors may have contributed to the low correlation values found in the granular unit level analysis.

Another limitation of this research is the limited timeframe used (21 January 2020 to 31 December 2020) where SARS-Cov-2 VOCs (e.g. Delta, Omicron) with much higher transmissibility are not present yet. Stringency Index (SI) which was primarily used for analysis in this study may not be the sole contributor in the COVID-19 chaotic system. As other studies have pointed out, COVID-19 transmissions may vary with mobility or human physical activity [36] in addition to the country’s socio-economic capabilities which could affect mass testing, contract tracing and timing of government response. We believe that an accurate prediction method can be accomplished using finer and highly specific SIs as one of the key variables in predicting COVID-19 cases. The effect of population density, its potential influence on the SI and consequent impact on COVID-19 interventions should be considered in the design of future models. The application of chaotic dimensions could lead to the creation of more accurate and highly specific COVID-19 prediction models which hopefully will influence decision-making and ultimately result to better policies and interventions equally advantageous to both the country’s economy and health care system.
